# Positron Emission Tomography/Magnetic Resonance Imaging Radiomics in Predicting Lung Adenocarcinoma and Squamous Cell Carcinoma

**DOI:** 10.3389/fonc.2022.803824

**Published:** 2022-02-03

**Authors:** Xin Tang, Jiangtao Liang, Bolin Xiang, Changfeng Yuan, Luoyu Wang, Bin Zhu, Xiuhong Ge, Min Fang, Zhongxiang Ding

**Affiliations:** ^1^ The Fourth Clinical College, Zhejiang Chinese Medical University, Hangzhou, China; ^2^ Department of Radiology, Hangzhou Wuyunshan Hospital (Hangzhou Health Promotion Research Institute), Hangzhou, China; ^3^ Department of Radiology, Hangzhou Universal Medical Imaging Diagnostic Center, Hangzhou, China; ^4^ Department of Radiology, Zhejiang Quhua Hospital, Quzhou, China; ^5^ Centre for Cognition and Brain Disorders, Hangzhou Normal University, Hangzhou, China; ^6^ Department of Radiology, Key Laboratory of Clinical Cancer Pharmacology and Toxicology Research of Zhejiang Province, Affiliated Hangzhou First People's Hospital, Cancer Center, Zhejiang University School of Medicine, Hangzhou, China

**Keywords:** lung, cancer, PET/MRI, radiomic, adenocarcinoma, squamous

## Abstract

**Objective:**

To investigate the diagnostic value of positron emission tomography (PET)/magnetic resonance imaging (MRI) radiomics in predicting the histological classification of lung adenocarcinoma and lung squamous cell carcinoma.

**Methods:**

PET/MRI radiomics and clinical data were retrospectively collected from 61 patients with lung cancer. According to the pathological results of surgery or fiberscope, patients were divided into two groups, lung adenocarcinoma and squamous cell carcinoma group, which were set as positive for adenocarcinoma (40 cases) and negative for squamous cell carcinoma (21 cases). The radiomics characteristics most related to lung cancer classification were calculated and selected using radiomics software, and the two lung cancer groups were randomly assigned into a training set (70%) and a test set (30%). Maximum relevance and minimum redundancy (mRMR) and least absolute shrinkage and selection operator (LASSO) methods in the uAI Research Portal software (United Imaging Intelligence, China) were used to select the desired characteristics from 2600 features extracted from MRI and PET. Eight optimal features were finally retained through 5-fold cross-validation, and a PET/MRI fusion model was constructed. The predictive ability of this model was evaluated by the difference in area under the curve (AUC) obtained from the receiver operating characteristic (ROC) curve.

**Results:**

AUC of PET/MRI model for the training group and test group were 0.886 (0.787-0.985) and 0.847 (0.648-1.000), respectively. PET/MRI radiomics features revealed different degrees of correlation with the classification of lung adenocarcinoma and squamous cell carcinoma, with significant differences.

**Conclusion:**

The prediction model constructed based on PET/MRI radiomics features can predict the preoperative histological classification of lung adenocarcinoma and squamous cell carcinoma without seminality and repeatability. It can also provide an objective basis for accurate clinical diagnosis and individualized treatment, thus having important guiding significance for clinical treatment.

## Introduction

In 2020, there were 19.29 million new cancer cases and 9.96 million cancer-related deaths worldwide, among which lung cancer accounted for 2.2 million (11.4%) and 1.8 million (18%), respectively. Thus, lung cancer has become the second most common cancer and the leading cause of death globally. In addition, lung cancer occurs more frequently in men with the highest incidence and mortality in males compared to other tumors (1). Lung cancer is divided into adenocarcinoma, squamous cell carcinoma, small cell carcinoma, etc., of which lung adenocarcinoma and squamous cell carcinoma are the main types, accounting for about 75% (2, 3). The treatment methods vary for different pathological types of lung cancer, and early diagnosis is of great significance for the diagnosis, overall treatment, and personalized treatment of patients with lung cancer.

Over recent years, the diagnosis and treatment of lung cancer have been further improved by the integration of radiomics, molecular biology, clinical and other disciplines. With the progress of imaging technology and the continuous development of drugs, especially the popularization and application of PET/CT and PET/MRI molecular radiomics technology, the performance level of clinical diagnosis and treatment efficacy evaluation of lung cancer have been greatly advanced. Sepehri et al. (4) found that the PET/CT radiomics-based model outperformed the standard clinical staging by retrospectively analyzing 138 patients with stage II-III non-small cell lung cancer. Ehman et al. (5) found that PET/CT had superiority in terms of use opportunity, application cost, examination speed and clinical awareness, but PET/MRI produced less radiation and was more advantageous in the detection of soft tissue tumors. In addition, for the staging of breast cancer, compared to PET/CT, PET/MRI can better distinguish the invasion of chest wall, diaphragm and mediastinum/distant soft tissues, which affected the TMN staging. As a result of the fusion of the metabolic information by PET with the high soft-tissue resolution and functional information by MRI, PET/MRI has gained more advantages in detecting primary soft tissue lesions, histopathological classification, TMN staging, prognosis prediction, efficacy evaluation, and recurrence detection. At the same time, as the fusion radiomics can determine the accurate location of the lesion and the anatomical relationship with the surrounding tissues, it has obvious advantages in determining the biological target area for lung cancer radiotherapy and formulating the extent of surgical resection. Thus, in the treatment of lung cancer, PET/MRI can be used for early observation of the tumor’s response to treatment, timely adjustment and optimization of the treatment plan, avoidance of ineffective treatment or toxic side effects, gaining treatment time for patients, improving the therapeutic effect, prolonging the survival time of patients and improving the quality of life.

The current diagnosis and treatment of lung cancer still mainly rely on the subjective experience of physicians and clinicians, and there is a lack of systematic analysis of the data information generated by radiomics examinations. Needle biopsy is the gold standard for pathological diagnosis of patients; still, it is invasive, reproducible, has potential complications, and is difficult to perform when the lesion is deep or adjacent to blood vessels. Therefore, this method has certain limitations and may even lead to fatal outcomes (3). However, radiomics methods use automated data characterization algorithms to transform medical images into high-resolution graphics, excavate feature spatial data, and quantify lesion morphological characteristics and internal heterogeneity (6–9). Deep mining of radiomics data can obtain many quantitative radiomics characteristics that the human eye cannot perceive.

This study aimed to find new radiomics quantitative parameters for histological classification of lung adenocarcinoma and squamous cell carcinoma based on PET/MRI radiomics method, construct a prediction model, and explore the diagnostic value of this technique in predicting the classification of lung adenocarcinoma and squamous cell carcinoma without seminality.

## Materials and Methods

### Subjects

A total of 61 patients with lung adenocarcinoma or squamous cell carcinoma confirmed by surgery or puncture, including 40 with lung adenocarcinoma and 21 with squamous cell carcinoma, who were initially diagnosed by PET/MRI examination in Hangzhou Universal Medical Imaging Diagnostic Center between October 2018 and August 2021 were retrospectively included in the study. The research protocol met the requirements of medical ethics (Scientific Research Medical Ethics, No. 2021-008), and all methods were implemented in accordance with the Declaration of Helsinki.

Inclusion criteria were the following: all patients underwent PET/MR examination before treatment and were pathologically confirmed to have adenocarcinoma or squamous cell carcinoma; no chemotherapy or radiotherapy and surgical anti-tumor therapy were performed; clear whole-body and chest PET/MR could be obtained before treatment; PET/MR examination was performed 40 – 60 min after injection of 18F-fluorodeoxyglucose (18F-FDG).

Exclusion criteria were: patients whose PET/MRI image failed to meet the diagnostic criteria (such as obvious metal or motion artifact); patients with contraindications to MRI examination or inability to tolerate the examination; patients with a history of other thoracic malignant tumors or other systemic malignancies; patients who had received any form of treatment before PET/MR examination (such as radiotherapy, chemotherapy, etc.); pathologically confirmed adenocarcinoma and squamous cell carcinoma of other histopathological types.

### Instruments and Equipments

Imaging data were acquired using integrated time-of-flight (TOF) PET/MR from GE (GE SIGNA, WI, USA). The system consisted of a PET detector with TOF technology (TOF-PET) and the latest generation of 750W 3.0T magnetic resonance. The TOF-PET detector is constructed with a state-of-the-art solid-phase array photoelectric converter (SiPM) and a new generation of LBS crystal. Simultaneous PET and MRI scanning were performed with the thinnest acquisition slice thickness of 2.8 mm (Transverse FOV: 60 cm, axial FOV: 25 cm, transverse resolution (1 cm from the center): 4.2 mm, axial resolution (1 cm from the center): 5.8 mm, temporal resolution: 385 ps, energy resolution: 11%, and sensitivity: 21 cps/kBq).

### Patients Preparation

Patients were required to fast for more than 6 hours, and the blood glucose concentration was controlled to be < 7.8 mmol/L before injection of ^18^F-FDG. On the examination day, patients wore clothes that did not have any accessories or were easy to take off. They were injected with ^18^F-FDG at a dose of 3.7 Mbq/kg and underwent whole-body PET/MRI 40 min later. Written informed consent was obtained from all patients or legal guardians before the examination.

### PET/MRI Scan

The patient was placed in the supine position. After performing attenuation correction, whole-body PET/MR scans were performed from the top of the head to the middle of the femur, and if necessary, sweeping to the sole of the foot. A total of 5 – 6 beds were collected, with an acquisition time of 6 minutes per bed. PET images was acquired and reconstructed using 3D mode, TOF technique, and point spread function (PSF) with ordered subset expectation maxima (OSEM) algorithm, which used two iterations, 28 subsets, and a 5 mm Gaussian post-processing filter with a 192 × 192 matrix. PET data acquisition was performed during a whole-body MRI examination. A regional PET/MR scan of the chest was then carried out, ranging from the apex to the base of the lung, and radiomics were obtained using dedicated MRI coils for the chest region, resulting in whole-body and regional PET, MRI, and PET/MR fusion radiomics. All data were acquired from the same PET/MR instrument. MRI sequences included LAVA-Flex T1WI, fs-PROPELLER T2WI, DWI (b = 800 mm^2^/s), and coronal fs-PROPELLER T2WI. In this study, chest local Axial T2WI radiomics and PET radiomics were selected as radiomics feature extraction sequences (10, 11).

### Radiomics Data Processing

Conjoined uAI Research Portal software (United Imaging Intelligence, China) that was embedded into the widely used package-PyRadiomics (https://pyradiomics.readthedocs.io/en/latest/index.html) was used for radiomics analysis on the region of interest (ROI) of the subject’s primary tumor. The workflow of radiomics mainly included the following steps: lesion segmentation, feature extraction, feature selection, and machine learning modeling (12–15).

### Lesion Segmentation

**Figure 1:**
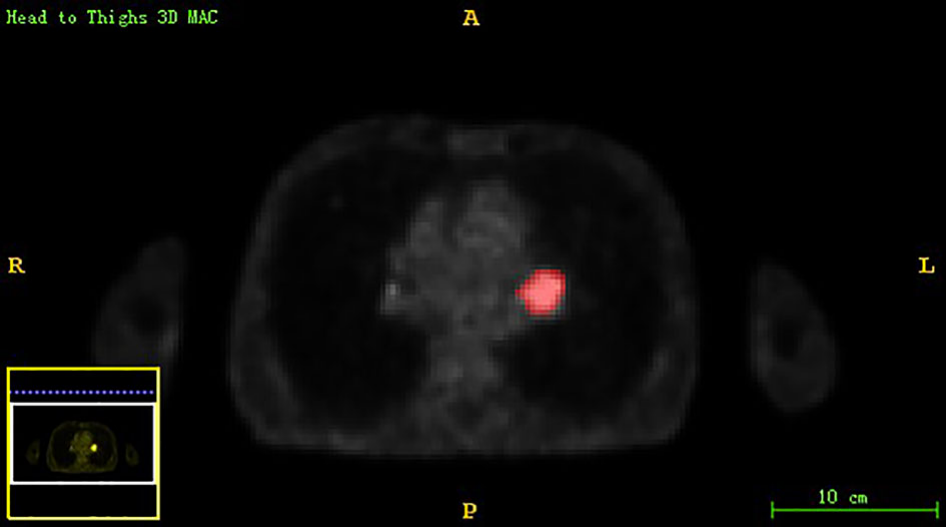
The lesion ROIs of axial PET sequence.

Chest PET and MRI data in DICOM format were imported into ITK-SNAP software (http://www.itksnap.org), which was used to delineate the region of interest (ROI) of the patient on PET and MRI axial data, manually delineate along the edge of the primary tumor of lung cancer, exclude adjacent normal tissues and lymph nodes, overlap the segmentation boundaries of PET and MRI data, and finally export the three-dimensional segmentation results obtained by PET and MRI radiomics sequentially into the original map and the corresponding ROI map (**Figures 1** **–** **4**). The segmentation result was saved as nii file. Two radiologists with 15 to 20 years of experience in thoracic PET/MR diagnosis simultaneously segmented ROIs on MRI and PET images of the primary lesion to obtain the corresponding ROI segmented graphics, respectively. When the results were inconsistent, the third radiologist with twenty years of experience performed ROI delineation again and checked until the results were unified.

**Figure 2:**
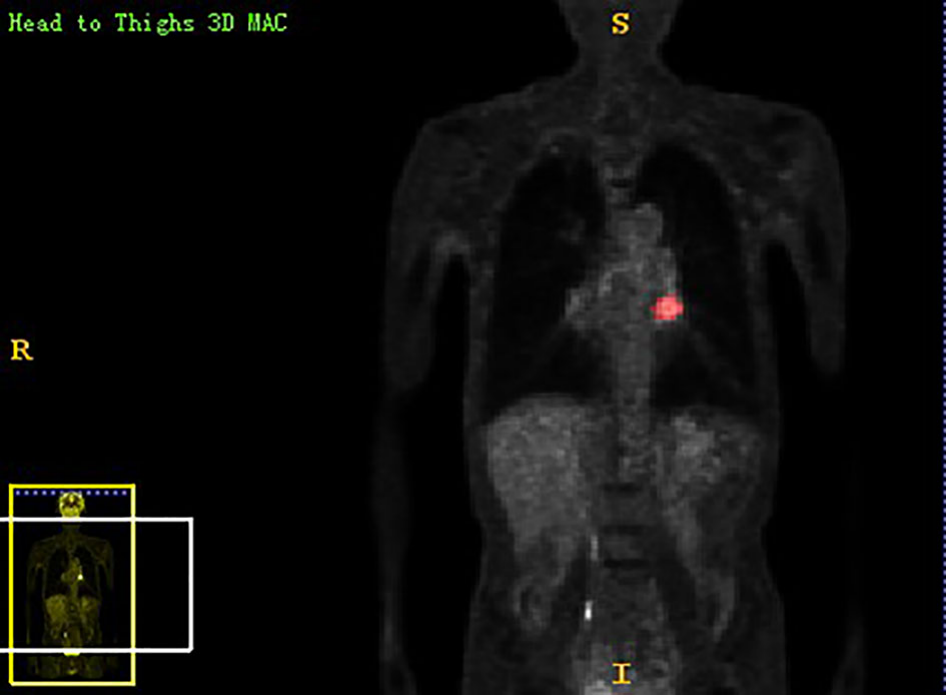
The lesion ROIs of coronal PET sequence.

**Figure 3:**
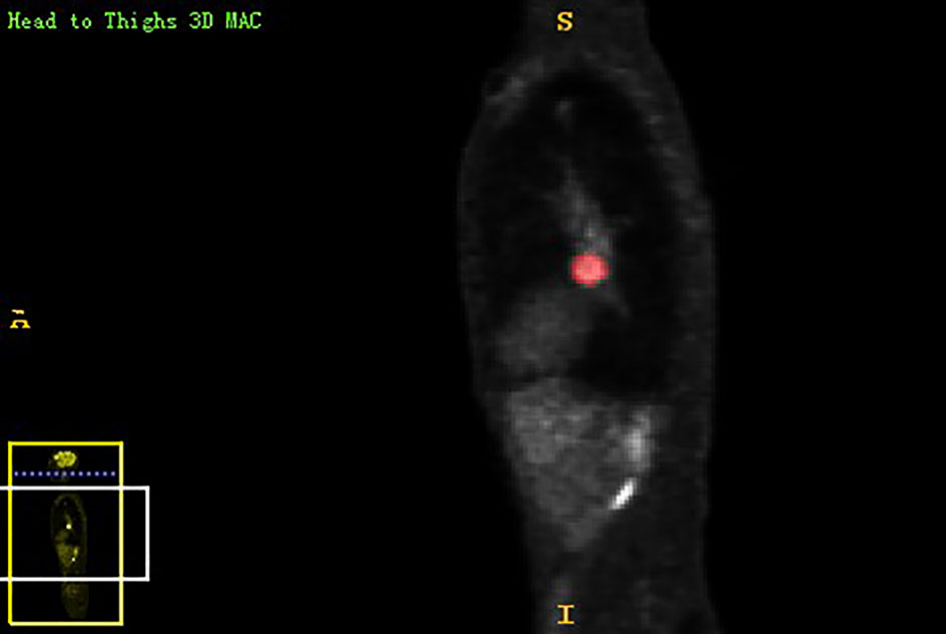
The lesion ROIs of sagittal PET sequence.

**Figure 4:**
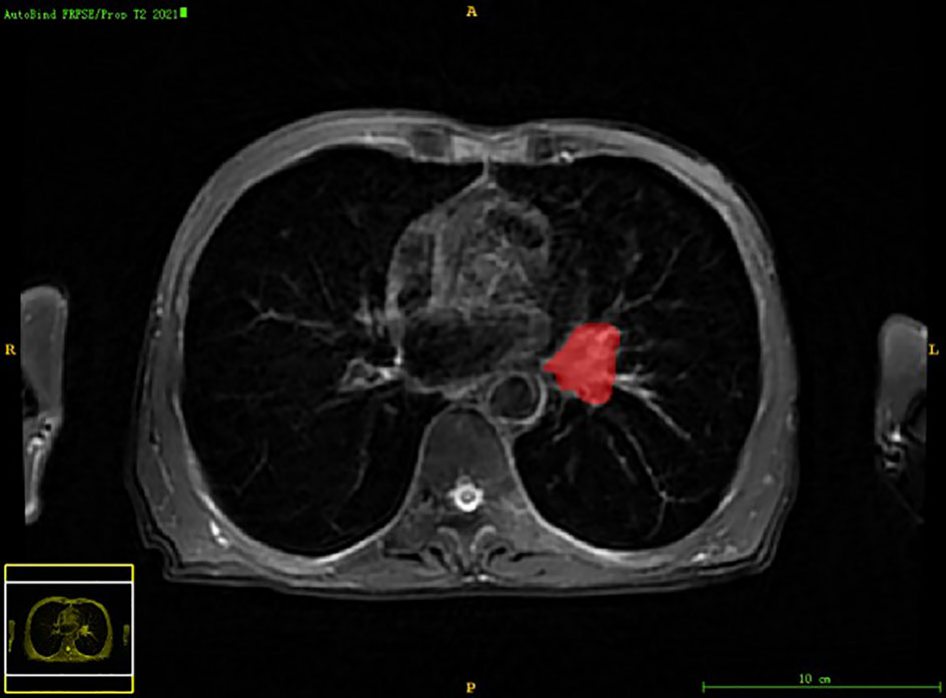
The lesion ROIs of axial MRI sequence.

### Feature Extraction

All ROI data and the original images of PET and MRI were imported into the uAI Research Portal software in batch.

### Feature Selection

#### Data Import

The radiomics of PET and MRI were imported into the R software (version 4.0.5, http://www.Rproject.org) for feature selection.

#### Feature Selection

The patients were randomly assigned into a training set (70%) and a test set (30%) (12, 16, 17). We used two feature selection methods, mRMR and LASSO, to select the features. Firstly, mRMR was performed to eliminate the redundant and irrelevant features; then LASSO was conducted to choose the optimized subset of features to construct the final model.

**Figure 5:**
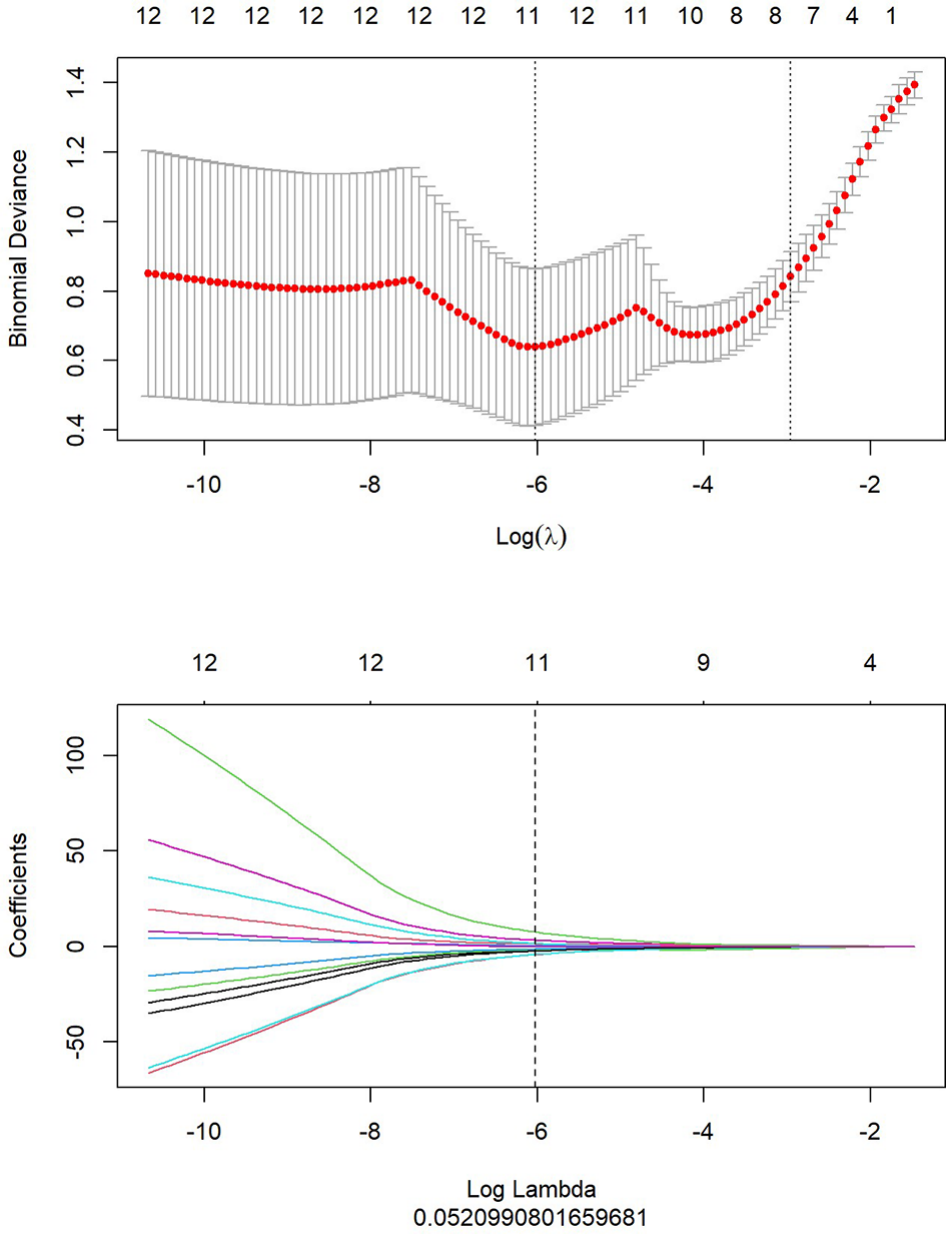
LASSO analysis of PET/MRI.

**Figure 6:**
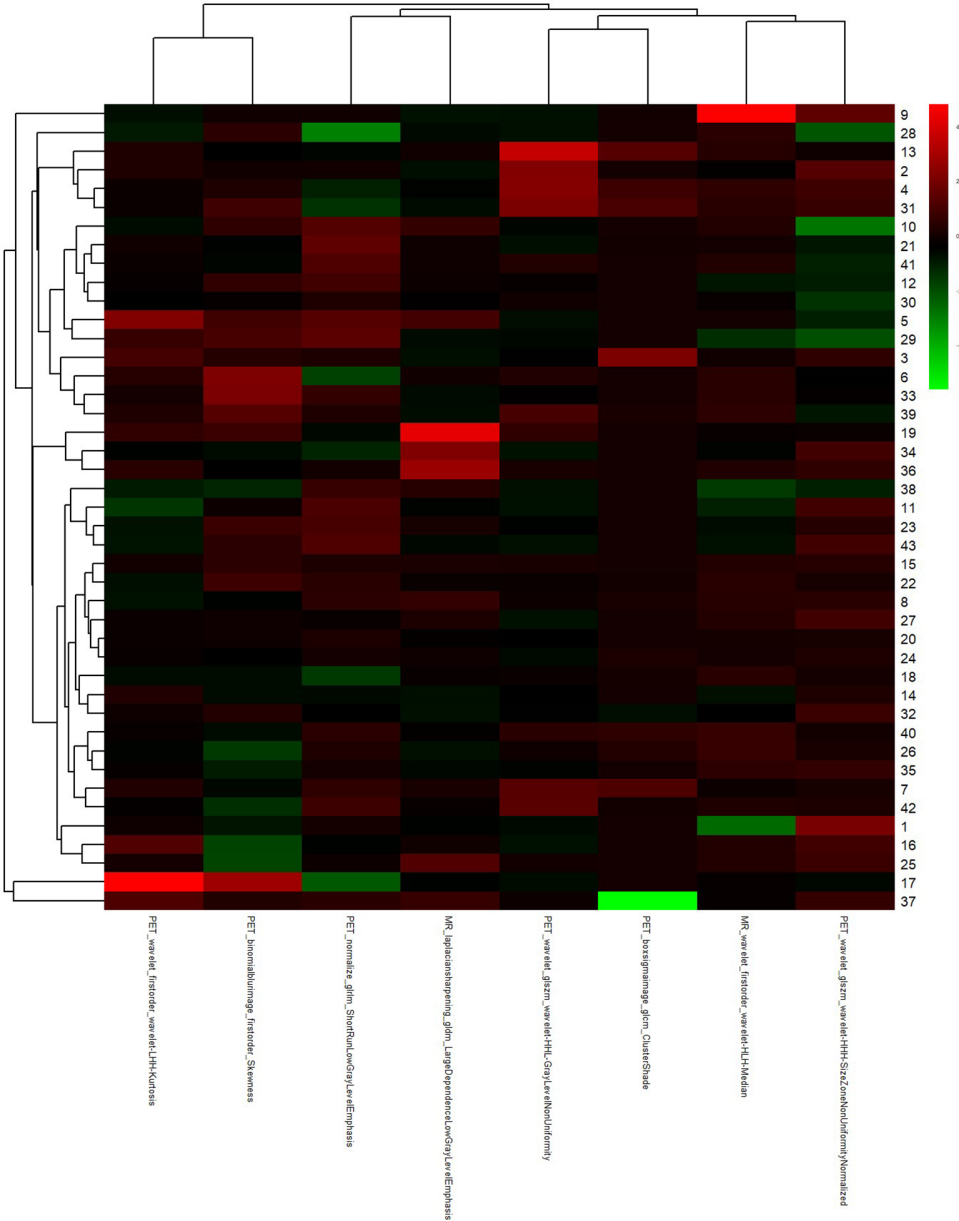
The most predictive feature subset of PET/MRI.

**Figure 7:**
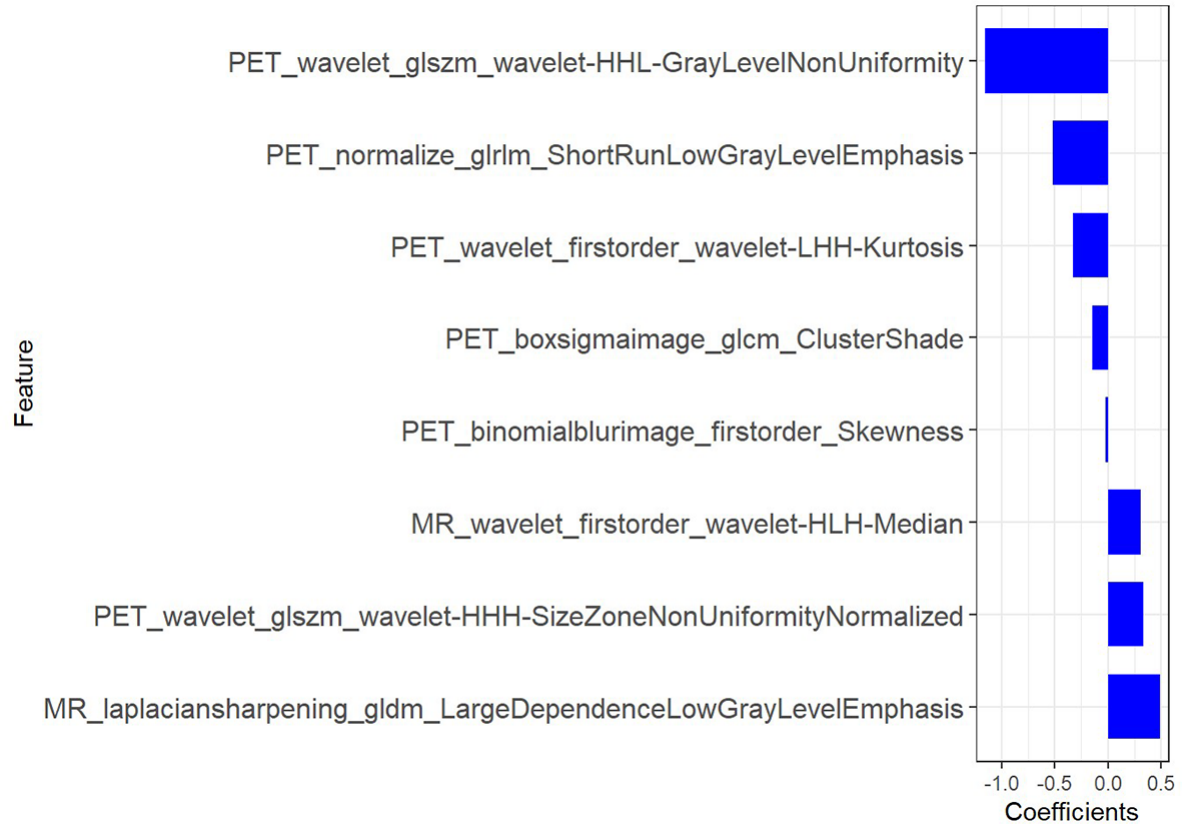
The radiomic coefficients of each feature in the most predictive feature subset in PET/MRI radiomics signature construction.

1. LASSO analysis included choosing the regular parameter λ, determining the number of the feature (**Figure 5**). After the number of feature was determined, the most predictive feature subset was chosen and the corresponding coefficients were evaluated (**Figure 6**).

2. After the number of feature was determined, the most predictive feature subset was chosen and the corresponding coefficients were evaluated (**Figure 7**).

The radiomic signature (Rad score) was calculated by summing the selected texture features, which were weighted by their respective coefficients. All rad scores between lung adenocarcinoma and squamous cell carcinoma group were compared in the training set and test set respectively.

The final formula for the PET/MRI rad score was: “Radscore=-1.161*PET_wavelet_glszm_wavelet-HHL-GrayLevelNonUniformity+-0.147*PET_boxsigmaimage_glcm_ClusterShade+-0.516*PET_normalize_glrlm_ShortRunLowGrayLevelEmphasis+0.311*MR_wavelet_firstorder_wavelet-HLH-Median+-0.332*PET_wavelet_firstorder_wavelet-LHH-Kurtosis+0.336*PET_wavelet_glszm_wavelet-HHH-SizeZoneNonUniformityNormalized+0.491*MR_laplaciansharpening_gldm_LargeDependenceLowGrayLevelEmphasis+-0.021*PET_binomialblurimage_firstorder_Skewness + 0.449”

#### Radiomics Validation

We used ROC analysis to evaluate the performance of the model (**Figure 10**).

#### Nomogram Building

### Statistical Analysis

Statistical comparisons of gender and age were performed using SPSS software (version 26). In addition, feature selection and radiomics signature construction and validation were conducted with R software. The statistical significance was set at a *P*-value of 0.05 with two-tailed analyses (3, 18). Feature extraction ROC measured the evaluation consistency between radiologists using the inter-correlation coefficients (ICC). All statistical methods of the radiomics analysis were conducted with uAI Research Portal software and R software.

## Results

In this study, the ICC value of > 0.86, which was considered to be in good agreement of the ROC. We selected ROC results from the senior radiologist to extract features.

### Comparison of Clinical Data

**Table 1** showed the results of statistical analysis of the demographic and clinical data. There were no statistically significant differences in age and gender between the lung adenocarcinoma and lung squamous cell carcinoma groups (**Table 1**).

**Table 1:** Summary of original data of cases.

Characteristic	Adenocarcinoma	squamous cell carcinoma	Statistical analysis	*P*-value
Gender			chi-square	*p* = 0.918
Male (case)	10	16		
Female (case)	30	5		
Age (years)	23-90	51-80	A two-sample T-test	*P* = 0.834

### Radiomics Analysis Results

**Figure 5** showed that top 20 imaging features were ranked and used as candidate features for LASSO regression analysis, according to the results with mRMR algorithm. In LASSO regression analysis, when Log (In) was -6 and Log Lambda was 0.521, the PET/MRI prediction model showed the best diagnostic performance, and the eight optimal imaging features were determined at this point.

**Figures 6**, **7** showed that eight characteristic parameters were obtained from the PET/MRI prediction model, which were PET_wavelet_glszm_wavelet-HHL-GrayLevelNonUniformity; PET_boxsigmaimage_glcm_ClusterShade; PET_normalize_glrlm_ShortRunLowGrayLevelEmphasis; MR_wavelet_firstorder_wavelet-HLH-Median; PET_wavelet_firstorder_wavelet-LHH-Kurtosis; PET_wavelet_glszm_wavelet-HHH-SizeZoneNonUniformityNormalized; MR_laplaciansharpening_gldm_LargeDependenceLowGrayLevelEmphasis; and PET_binomialblurimage_firstorder_Skewness.

**Figure 8:**
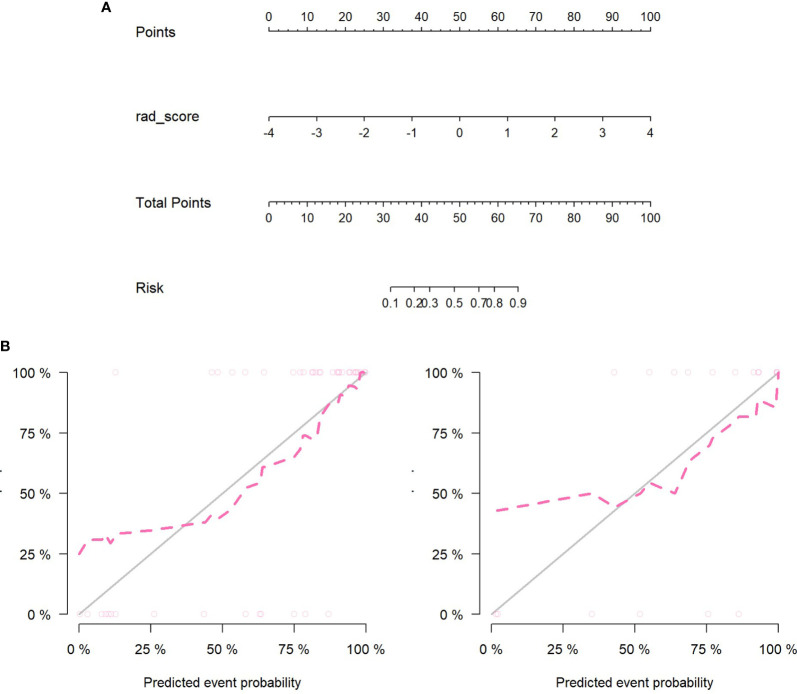
**(A)** Nomogram construction in the PET/MRI training set. **(B)** The left figure is the training set calibration curve; the right figure is the test set calibration curve; the solid black line represents the theoretical curve, and the red dashed line represents the deviation correction curve. The formula of PET/MRI nomoscore is defined as follows: “Nomoscore = (Intercept)*0.188+rad_score*1.648”.

**Figures 8A, B** showed that the predicted values of the training and test group were very close to the actual values, and they showed that the prediction ability of the nomogram was good.

**Figure 9:**
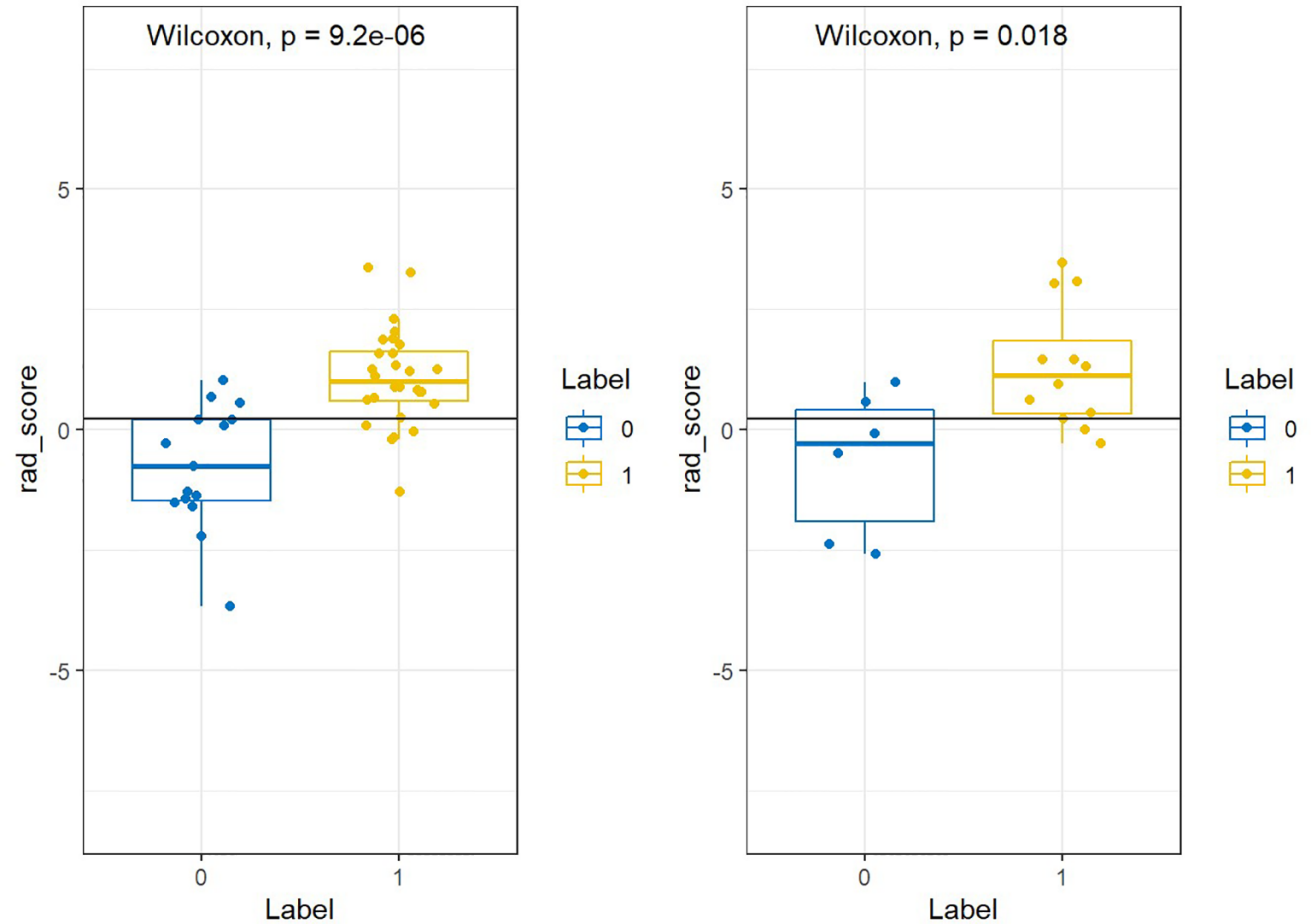
The label of PET/MRI.

**Figure 9** showed that labels “0” and “1” were added to the rad scores of the training and test groups, respectively, where adenocarcinoma was labeled “1” and scale-cell carcinoma was labeled “0”.

**Figure 10:**
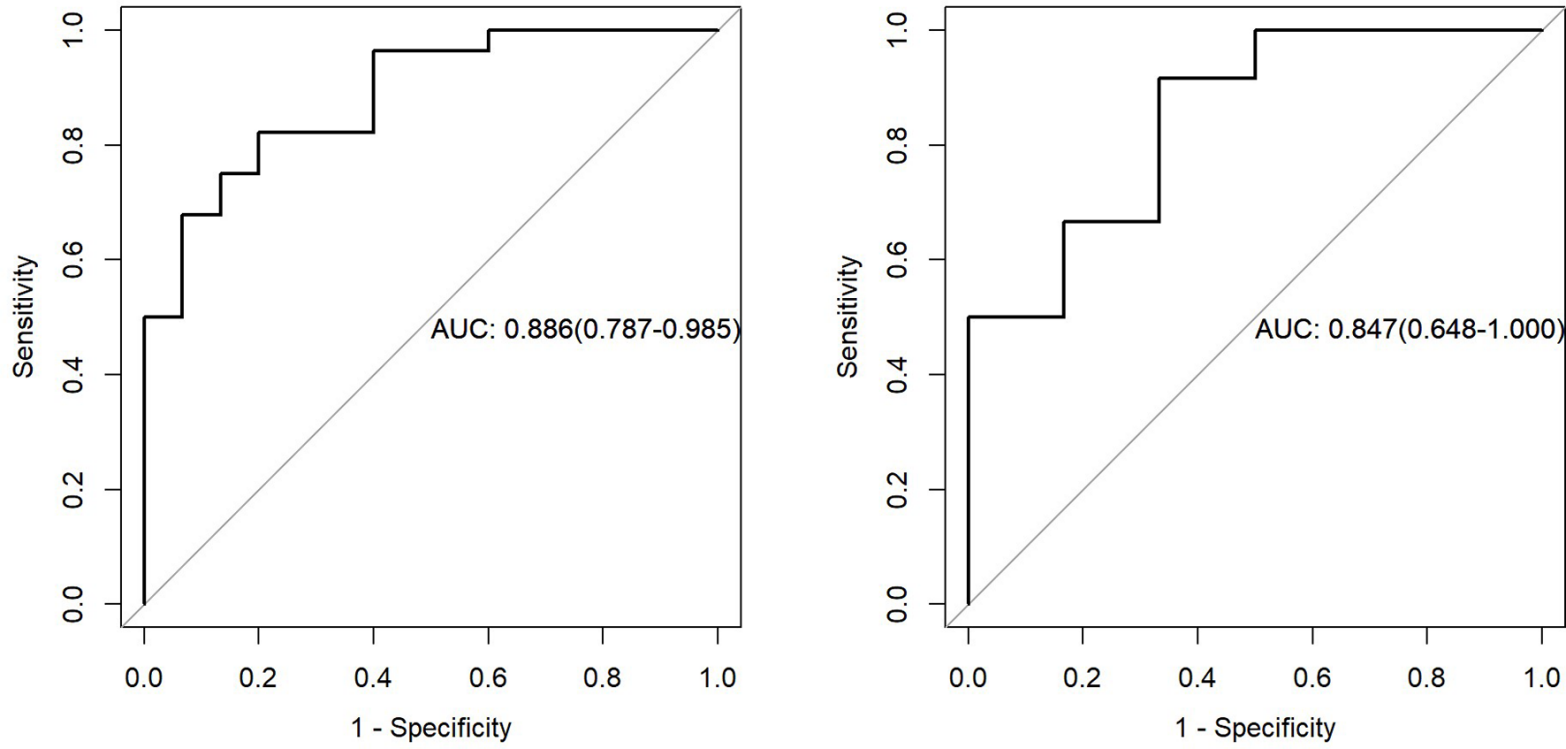
The left figure represents the AUC of PET/MRI model in the training set, and the right figure represents the AUC of PET/MRI model in the test set.

**Figure 10** showed that AUC of PET/MRI model in the training and test group was 0.886 (0.787-0.985) and 0.847 (0.648-1.000), respectively. Based on Youden Index, other parameters were calculated as follows (**Table 2**):

**Table 2:** Results of PET/MRI radiomics.

Group	Accuracy	Accuracy Lower	Accuracy Upper	Sensitivity	Specificity	Pos.Pred.Value	Neg.Pred.Value
Training	0.814	0.666	0.916	0.800	0.821	0.706	0.885
Test	0.833	0.586	0.964	0.667	0.917	0.800	0.846

**Figure 11:**
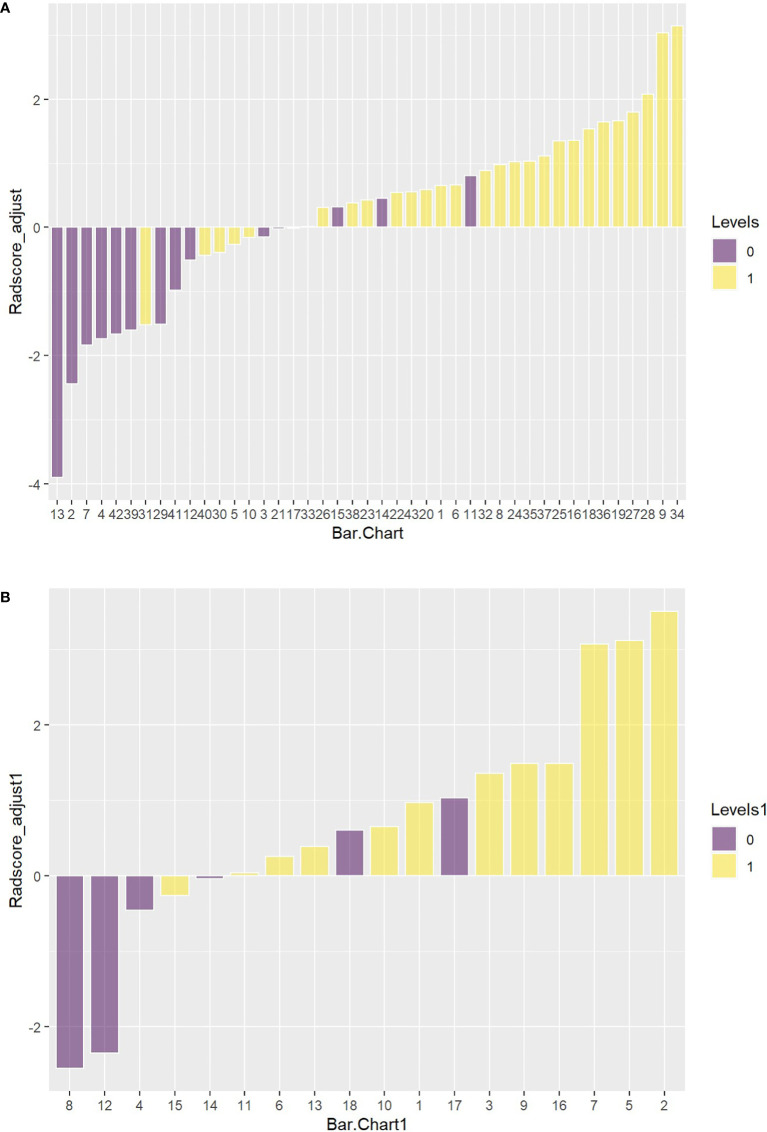
**(A)** The Hosmer-Lemeshow result for the PET/MRI model in the training set. **(B)** The Hosmer-Lemeshow results for PET/MRI model in the test set.

**Figures 11A, B** showed the Hosmer-Lemeshow test result.

**Figure 12:**
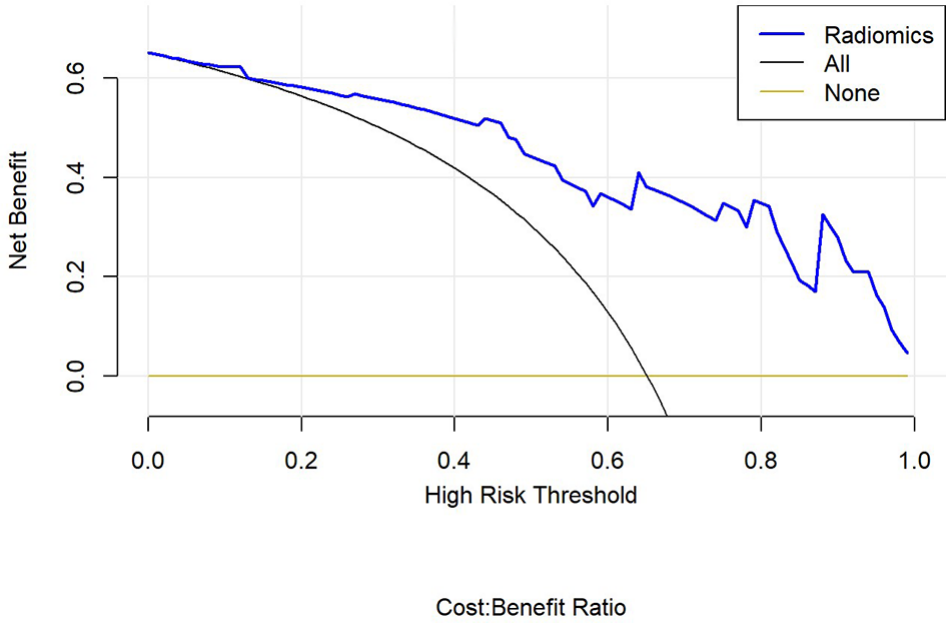
Decision curve analysis for different variables in PET/MRI models.

Finally, we used decision curve to evaluate the clinical usefulness of the model (**Figure 12**).

## Discussion

In this study, PET/MRI prediction models established based on chest MRI and PET radiology were used to analyze 40 patients with lung adenocarcinoma and 21 patients with squamous cell carcinoma, and finally 8 optimal characteristic parameters were obtained, of which 3 belonged to intensity characteristics and 5 belonged to texture characteristics, 6 characteristics were from PET radiology and 2 characteristics were from MRI radiology. These results indicated that texture characteristics were more related to lung adenocarcinoma and lung squamous cell carcinoma classification, of which the characteristics with the highest feature weight ratio was wavelet GLSZM-HHL-GLNU, indicating that it had the most significant predictive effect on NSCLC patient subtypes in PET/MRI models, and GLSZM was a standardized distribution of regional counts relative to gray values. The lower the value was, the more uniform the intensity value became. Moreover, GLSZM is negatively correlated with survival and helps identify hypoxic or necrotic areas with poor prognosis. Yang et al. (19) showed that gray level run length matrix (GLRLM) and wavelet characteristics were related to the survival time of lung cancer. Additionally, the AUC of the PET/MRI model in the training and test set were 0.886 (0.787 – 0.985) and 0.847 (0.648 – 1.000), respectively, which were very close. The results showed that the PET/MRI prediction model had good fit, good consistency and stability, and also showed that the PET/MRI prediction model could effectively and non-invasively classify the pathological types of lung adenocarcinoma and lung squamous cell carcinoma. The diagnostic value of PET/MR prediction model in the training and test set suggested that the model constructed based on PET/MRI radiomics characteristics had a high predictive value for preoperative pathological classification of lung adenocarcinoma and squamous cell carcinoma, providing an objective basis for accurate clinical diagnosis and individualized treatment, and having an important guiding significance for clinical treatment. The Hosmer-Lemeshow test indicated that the PET/MRI prediction model could effectively differentiate the pathological subtypes of lung adenocarcinoma from lung squamous cell carcinoma. Decision curve analysis of different variables for clinical application of PET/MRI model showed that the net benefit of the PET/MRI radiomics model under different threshold probabilities outweighed the clinical variables. We found that these curves could further reflect the clinical utility and the higher predictive efficiency of PET/MRI radiomics models.

Coroller et al. (20) analyzed the CT radiomics of 85 patients with locally advanced NSCLC and extracted the radiomics parameters of lymph nodes and primary tumors using radiomics methods. They reported that the phenotypic information of lymph nodes was more effective than that of primary tumors in predicting the pathological responses. Kirienko et al. (21) retrospectively analyzed the radiomics data of PET and CT of 534 lung lesions cases and found that the texture characteristics of PET radiomics using the method of linear discriminant analysis could distinguish primary lung cancer from metastatic tumors (AUC > 0.90), and could classify the histological subtypes of primary lung cancer (AUC 0.61, 0.97). Other studies have also evaluated the relationship between tumor subtypes, histopathological grades, diagnosis, treatment, and prognosis of lung cancer and radiomics characteristics. Orlhac and colleagues (22) compared the texture characteristics of adenocarcinoma and squamous cell carcinoma and showed differences in most texture characteristics, while squamous cell carcinoma had lower homogeneity and higher entropy, reflecting its higher heterogeneity than adenocarcinoma. Yang et al. (23) retrospectively analyzed PET/CT radiology data of 315 NSCLC patients and found that the radiological nomograms based on 18F-FDG PET/CT rad score and clinicopathological factors had a good predictive performance for survival outcomes, providing feasible and practical guidance for individualized management of NSCLC patients. Szyszko et al. (24) and Grosse et al. (25) reported that the main advantages of PET/MRI were the reduction of radiation dose and the improvement of anatomical details in soft tissue areas, making it suitable for pediatric patients and patients requiring repeated radiomics. Hyun et al. (26) established a PET prediction model that successfully predicted histological subtypes of lung cancer and found that the logistic regression model outperformed all other classifiers (AUC = 0.859, accuracy = 0.769). In the present study, we used mRMR and LASSO methods to construct a combined PET/MRI prediction model based on chest MRI and PET, and showed that the results of the training group, with an AUC of 0.886 and an accuracy of 0.814. We concluded that the PET/MRI prediction model was superior to the PET prediction model in terms of predicting the pathological types of lung adenocarcinoma and squamous cell carcinoma, and the PET/MRI radiomics might be more helpful for clinicians to improve the histopathological diagnosis. As a non-invasive radiomics method, PET/MRI can significantly reduce the radiation dose compared with PET/CT and provide morphological, functional, and molecular radiomics information of the tumor in one examination. Compared with histopathological and genetic testing methods, PET/MR examination can overcome sampling deviations and complications caused by biopsy. It can also provide more comprehensive and accurate information in predicting biomarkers. This study showed that PET/MRI could be used for non-seminal evaluation and prediction of lung adenocarcinoma and squamous cell carcinoma, which was conducive to develop a specific and individualized treatment plan for lung cancer patients in clinical practice.

The present study had some limitations. Firstly, this was a single-center retrospective study with a limited sample size. Furthermore, the cost of PET/MRI was too expensive and PET/MRI examination required the use of multi-sequence MR radiomics. The examination time was too long, the patient could not fully cooperate statically, and the rapidly changing gradient of MRI produced great noise, resulting in the inability to directly assess tissue density, especially for the lung and bone. Therefore, an accurate attenuation correction map was obtained, and the interference caused by different radiomics acquisition parameters and respiratory motion displacement might reduce the diagnostic accuracy of the model. In the future, more data on lung cancer patients’ samples can be obtained, new MRI examination sequences can be developed, respiratory and motion artifacts can be improved, more comfortable bed surfaces and audio and video equipments for PET/MRI machines can be improved, and examination time can be shortened without affecting radiomics quality. It is necessary to select appropriate machine learning algorithms, build multi-modal and multi-center cooperation, improve the prediction efficiency, minimize the risk of overfitting, perform more refined sample data processing, and construct artificial intelligence classification models with higher complexity that may have an important role in the accurate classification and prediction of lung adenocarcinoma and lung squamous cell carcinoma. At present, the advantages of PET/MRI do not exceed their disadvantages. Nevertheless, PET/MRI is equivalent or complementary to PET/CT for lung tumor detection. At the same time, the radiomics research is still in its infancy, and there is a lot of possibility for future development.

With the development of artificial intelligence and radiomics, a multi-modal combination of clinical, radiomics, and pathological data will be adopted. Therefore, it is believed that imaging-based PET/MRI prediction for lung cancer classification will have a promising future in the clinical auxiliary diagnosis of lung cancer.

## Data Availability Statement

The original contributions presented in the study are included in the article/supplementary material. Further inquiries can be directed to the corresponding author.

## Ethics Statement

The studies involving human participants were reviewed and approved by Scientific Research Medical Ethics, No. 2021-008. The patients/participants provided their written informed consent to participate in this study.

## Author Contributions

Guarantor of integrity of the entire study: ZD. Study concepts: XT, JL, BX. Study design: XT, JL, CY. Literature research: XT, BX. Data acquisition: XT, JL, BZ, MF, XG. Statistical analysis: LW. Manuscript preparation: XT, JL. Manuscript editing: XT. Manuscript review: ZD. All authors contributed to the article and approved the submitted version.

## Funding

National Natural Science Foundation of China (81871337), Natural Science Foundation of Zhejiang Province (Y22H185692, LY16H180007), and Science Foundation from the Health Commission of Zhejiang Province (A20200507).

## Conflict of Interest

The authors declare that the research was conducted in the absence of any commercial or financial relationships that could be construed as a potential conflict of interest.

## Publisher’s Note

All claims expressed in this article are solely those of the authors and do not necessarily represent those of their affiliated organizations, or those of the publisher, the editors and the reviewers. Any product that may be evaluated in this article, or claim that may be made by its manufacturer, is not guaranteed or endorsed by the publisher.

## References

[1] Sung H Ferlay J Siegel RL Laversanne M Soerjomataram I Jemal A. Global Cancer Statistics 2020: GLOBOCAN Estimates of Incidence and Mortality Worldwide for 36 Cancers in 185 Countries. CA Cancer J Clin (2021) 71(3):209–49. doi: 10.3322/caac.21660 33538338

[2] Travis WD. Pathology of Lung Cancer. Clin Chest Med (2011) 32(4):669–92. doi: 10.1016/j.ccm.2011.08.005 22054879

[3] Li H Gao L Ma H Arefan D He J Wang J. Radiomics-Based Features for Prediction of Histological Subtypes in Central Lung Cancer. Front Oncol (2021) 11:658887. doi: 10.3389/fonc.2021.658887 33996583 PMC8117140

[4] Sepehri S Tankyevych O Upadhaya T Visvikis D Hatt M Cheze Le Rest C. Comparison and Fusion of Machine Learning Algorithms for Prospective Validation of PET/CT Radiomic Features Prognostic Value in Stage II-III Non-Small Cell Lung Cancer. Diagn (Basel) (2021) 11(4):675. doi: 10.3390/diagnostics11040675 PMC8069690 33918681

[5] Ehman EC Johnson GB Villanueva-Meyer JE Cha S Leynes AP Larson PEZ. PET/MRI: Where Might it Replace PET/Ct? J Magn Reson Imaging (2017) 46(5):1247–62. doi: 10.1002/jmri.25711 PMC5623147 28370695

[6] Schacht DV Drukker K Pak I Abe H Giger ML. Using Quantitative Image Analysis to Classify Axillary Lymph Nodes on Breast MRI: A New Application for the Z 0011 Era. Eur J Radiol (2015) 84(3):392–7. doi: 10.1016/j.ejrad.2014.12.003 PMC4628184 25547328

[7] Andersen MB Harders SW Ganeshan B Thygesen J Torp Madsen HH Rasmussen F. CT Texture Analysis can Help Differentiate Between Malignant and Benign Lymph Nodes in the Mediastinum in Patients Suspected for Lung Cancer. Acta Radiol (2016) 57(6):669–76. doi: 10.1177/0284185115598808 26271125

[8] Kunimatsu A Kunimatsu N Kamiya K Watadani T Mori H Abe O. Comparison Between Glioblastoma and Primary Central Nervous System Lymphoma Using MR Image-Based Texture Analysis. Magn Reson Med Sci (2018) 17(1):50–7. doi: 10.2463/mrms.mp.2017-0044 PMC5760233 28638001

[9] Zhou W Zhang L Wang K Chen S Wang G Liu Z. Malignancy Characterization of Hepatocellular Carcinomas Based on Texture Analysis of Contrast-Enhanced MR Images. J Magn Reson Imaging (2017) 45(5):1476–84. doi: 10.1002/jmri.25454 27626270

[10] Fan L Sher A Kohan A Vercher-Conejero J Rajiah P. PET/MRI in Lung Cancer. Semin Roentgenol (2014) 49(4):291–303. doi: 10.1053/j.ro.2014.07.002 25498226

[11] Feng Q Liang J Wang L Niu J Ge X Pang P. Radiomics Analysis and Correlation With Metabolic Parameters in Nasopharyngeal Carcinoma Based on PET/MR Imaging. Front Oncol (2020) 10:1619. doi: 10.3389/fonc.2020.01619 33014815 PMC7506153

[12] Chong H Gong Y Pan X Liu A Chen L Yang C. Peritumoral Dilation Radiomics of Gadoxetate Disodium-Enhanced MRI Excellently Predicts Early Recurrence of Hepatocellular Carcinoma Without Macrovascular Invasion After Hepatectomy. J Hepatocell Carcinoma (2021) 8:545–63. doi: 10.2147/JHC.S309570 PMC8200148 34136422

[13] Wang W Gu D Wei J Ding Y Yang L Zhu K. A Radiomics-Based Biomarker for Cytokeratin 19 Status of Hepatocellular Carcinoma With Gadoxetic Acid-Enhanced MRI. Eur Radiol (2020) 30(5):3004–14. doi: 10.1007/s00330-019-06585-y 32002645

[14] Ji GW Zhu FP Xu Q Wang K Wu MY Tang WW. Machine-Learning Analysis of Contrast-Enhanced CT Radiomics Predicts Recurrence of Hepatocellular Carcinoma After Resection: A Multi-Institutional Study. EBioMedicine (2019) 50:156–65. doi: 10.1016/j.ebiom.2019.10.057 PMC6923482 31735556

[15] Kim J Choi SJ Lee SH Lee HY Park H. Predicting Survival Using Pretreatment CT for Patients With Hepatocellular Carcinoma Treated With Transarterial Chemoembolization: Comparison of Models Using Radiomics. AJR Am J Roentgenol (2018) 211(5):1026–34. doi: 10.2214/AJR.18.19507 30240304

[16] Wang XH Long LH Cui Y Jia AY Zhu XG Wang HZ. MRI-Based Radiomics Model for Preoperative Prediction of 5-Year Survival in Patients With Hepatocellular Carcinoma. Br J Cancer (2020) 122(7):978–85. doi: 10.1038/s41416-019-0706-0 PMC7109104 31937925

[17] Gu D Xie Y Wei J Li W Ye Z Zhu Z. MRI-Based Radiomics Signature: A Potential Biomarker for Identifying Glypican 3-Positive Hepatocellular Carcinoma. J Magn Reson Imaging (2020) 52(6):1679–87. doi: 10.1002/jmri.27199 32491239

[18] Feng Q Niu J Wang L Pang P Wang M Liao Z. Comprehensive Classification Models Based on Amygdala Radiomic Features for Alzheimer’s Disease and Mild Cognitive Impairment. Brain Imaging Behav (2021) 15(5):2377–86. doi: 10.1007/s11682-020-00434-z 33537928

[19] Yang L Yang J Zhou X Huang L Zhao W Wang T. Development of a Radiomics Nomogram Based on the 2D and 3D CT Features to Predict the Survival of non-Small Cell Lung Cancer Patients. Eur Radiol (2019) 29(5):2196–206. doi: 10.1007/s00330-018-5770-y 30523451

[20] Coroller TP Agrawal V Huynh E Narayan V Lee SW Mak RH. Radiomic-Based Pathological Response Prediction From Primary Tumors and Lymph Nodes in NSCLC. J Thorac Oncol (2017) 12(3):467–76. doi: 10.1016/j.jtho.2016.11.2226 PMC5318226 27903462

[21] Kirienko M Cozzi L Rossi A Voulaz E Antunovic L Fogliata A. Ability of FDG PET and CT Radiomics Features to Differentiate Between Primary and Metastatic Lung Lesions. Eur J Nucl Med Mol Imaging (2018) 45(10):1649–60. doi: 10.1007/s00259-018-3987-2 29623375

[22] Orlhac F Soussan M Chouahnia K Martinod E Buvat I. 18f-FDG PET-Derived Textural Indices Reflect Tissue-Specific Uptake Pattern in Non-Small Cell Lung Cancer. PloS One (2015) 10(12):e0145063. doi: 10.1371/journal.pone.0145063 26669541 PMC4682929

[23] Yang B Zhong J Zhong J Ma L Li A Ji H. Development and Validation of a Radiomics Nomogram Based on 18F-Fluorodeoxyglucose Positron Emission Tomography/Computed Tomography and Clinicopathological Factors to Predict the Survival Outcomes of Patients With Non-Small Cell Lung Cancer. Front Oncol (2020) 10:1042. doi: 10.3389/fonc.2020.01042 32766134 PMC7379864

[24] Szyszko TA Cook GJR. PET/CT and PET/MRI in Head and Neck Malignancy. Clin Radiol (2018) 73(1):60–9. doi: 10.1016/j.crad.2017.09.001 29029767

[25] Grosse J Dirk H. PET/CT Und PET/MRT Bei Tumoren Des Kopf-Hals-Bereichs” [PET/CT and PET/MRI in Head and Neck Cancer]. Laryngo-rhino-otologie (2020) 99(1):12–21. doi: 10.1055/a-1057-1244 31935754

[26] Hyun SH Ahn MS Koh YW Lee SJ. A Machine-Learning Approach Using PET-Based Radiomics to Predict the Histological Subtypes of Lung Cancer. Clin Nucl Med (2019) 44(12):956–60. doi: 10.1097/RLU.0000000000002810 31689276

